# Extended X-ray absorption fine structure of bimetallic nanoparticles

**DOI:** 10.3762/bjnano.2.28

**Published:** 2011-05-11

**Authors:** Carolin Antoniak

**Affiliations:** 1 Fakultät für Physik and Center for Nanointegration Duisburg–Essen (CeNIDE), Universität Duisburg–Essen, Lotharstr. 1, 47048 Duisburg, Germany

**Keywords:** bimetallic alloys, EXAFS, FePt, nanoparticles, wavelets, XAS

## Abstract

Electronic and magnetic properties strongly depend on the structure of the material, especially on the crystal symmetry and chemical environment. In nanoparticles, the break of symmetry at the surface may yield different physical properties with respect to the corresponding bulk material. A useful tool to investigate the electronic structure, magnetic behaviour and local crystallographic structure is X-ray absorption spectroscopy. In this review, recent developments in the field of extended X-ray absorption fine structure measurements and in the analysis methods for structural investigations of bimetallic nanoparticles are highlighted. The standard analysis based on Fourier transforms is compared to the relatively new field of wavelet transforms that have the potential to outperform traditional analysis, especially in bimetallic alloys. As an example, the lattice expansion and inhomogeneous alloying found in FePt nanoparticles is presented, and this is discussed below in terms of the influence of employed density functional theory calculations on the magnetic properties.

## Introduction

Since the discovery of X-rays in 1895 by Röntgen, the field of spectroscopy methods using this regime of the electromagnetic spectrum has reached a very important status nowadays, e.g., in material sciences, physics, chemistry, and biology. The advent of synchrotron radiation sources in the 1960s set a milestone in the improvement of the brilliance of X-ray radiation, i.e., of the number of emitted photons per second per unit solid angle in a narrow energy bandpass (usually 0.1%). The increase in average brilliance of X-rays available from artificial sources, from the first X-ray tubes to synchrotron radiation sources of the third generation, is a remarkable factor of about 10^13^. For next generation free electron lasers an additional increase in the peak brilliance by ten orders of magnitude is aimed for [1].

A detailed description of synchrotron radiation sources and optical devices can be found, e.g., in [2–6]. The interested reader may also be referred to [7], in which the electrodynamics behind synchrotron radiation are explained. Here we focus on state-of-the-art X-ray absorption spectroscopy (XAS) on 3rd generation synchrotron sources such as the ESRF and BESSY II.

In general, XAS deals with the excitation of core-level electrons, with their element-specific binding energies, by incident X-rays. After absorption of an X-ray photon, a core-hole remains at the former state of the photoelectron and there exist two relaxation channels for de-excitation of the atom: After the transition of another electron from an energetically higher level into the core-hole state, the resulting energy gain either drives the emission of a fluorescence photon or an Auger electron. The X-ray absorption can be measured by detection of the emitted fluorescence photons (fluorescence yield, FY) or by detection of the Auger and secondary electrons (electron yield, EY). For thin samples, the absorption can also be measured in transmission geometry by comparing the intensity of incident X-rays to the intensity of X-rays passed through the sample.

At low photoelectron energies, ranging from the absorption edge up to about 100 eV above, XAS is sensitive to the electronic structure around the absorbing atom and gives rise to the X-ray absorption near-edge structure (XANES). In the energy range above the XANES region – typically from 100 eV to 1000 eV above the absorption edge – the extended X-ray absorption fine structure (EXAFS) contains information about the type and distance of atoms in the local environment of the absorbing atom.

In the literature several examples can be found for EXAFS analysis on nanoparticle systems, e.g., Co [8], CdS [9], CdSe [10], SnO_2_ [11] and Au [12] nanoparticles, as well as Ag nanoparticles embedded in glass [13–14]. To discuss the advantages and possible drawbacks of EXAFS analysis in nanoparticulate systems, this paper is organised as follows: In the next few subsections, the basics of XANES and EXAFS are shortly summarised. The second section focuses on the EXAFS analysis either on the basis of standard Fourier transform (FT) methods or by using a wavelet transform (WT). As an example, recent results on FePt nanoparticles are presented after a short summary of different preparation methods. The EXAFS results are also discussed regarding the influence of local structure and composition on the magnetic properties in an alloy, before conclusions are given in the last section.

## Review

### X-ray absorption near-edge structure (XANES)

The XANES includes information about the density of states (DOS) of the absorbing atoms. More precisely, the XANES is connected to the unoccupied electronic DOS of the excited atom in the presence of a core-hole. In this section, the description of the XANES by the standard one-electron (quasiparticle) picture is briefly summarised. More sophisticated approaches based on the Bethe–Salpeter equation for a two-particle system in quantum field theory have also been developed [15–16]. However, since the quasiparticle approximation leads to a good agreement between theory and experiment in common cases, it is specifically presented below. A discussion as to why this simple approach works fairly well can be found in the work of J. J. Rehr [17].

As known from visible light, the intensity of X-rays after passing through matter of a certain thickness, *x*, obeys the Lambert–Beer law:

[1]
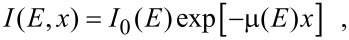


where *I*_0_ is the (energy dependent) intensity of incident X-rays and μ is the absorption coefficient. Since for photon energies below 20 keV the photoeffect dominates over Raleigh and Compton scattering, μ can be approximated by the photoabsorption coefficient, which is proportional to the absorption cross-section. The latter is given by the transition probability per unit time, *P*_fi_, from the initial state i to the final state f, normalised to the photon flux. *P*_fi_ can be described using Fermi’s Golden Rule in the one-electron approximation:

[2]
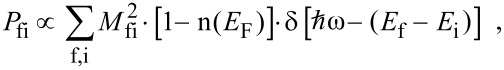


where 1−n(*E*_F_) is the density of unoccupied final states and the δ-function reflects the conservation of energy in the absorption process. The transition matrix element can be written within the electric dipole approximation (E1) as

[3]
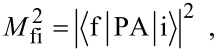


where P is the electron’s momentum operator and A is the electric field vector containing the polarisation of the X-rays. For this case, transitions are allowed according to the dipole selection rules

[4]
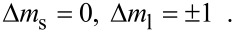


The spin rule Δ*m*_s_ = 0 reflects the fact that the electron transitions exclude a spin flip, and Δ*m*_l_ = ±1 is the Laporte rule [18]. Essentially, the Laporte rule states that transitions are forbidden between states that have the same symmetry with respect to the inversion operation, and it originates from a quantum mechanical selection rule which states that parity should be inverted during an electronic transition.

In the E1 approximation, electron transitions, e.g., from the 1s state (K absorption edge) to p states and from 2p states (L_3,2_ absorption edges) to d states, are described. In the electric quadrupole (E2) approximation, additional Laporte-forbidden electron transitions according to Δ*m*_l_ = ±2 are included, e.g., from s to d states. They are connected to a loss of symmetry that can occur for various reasons, e.g., by Jahn–Teller distortion or vibrational asymmetries in complexes. A short general description of the “appearance of ‘forbidden lines’ in spectra” can be found in [19] together with the discussion of higher order terms, i.e., the octopole. A more elaborate presentation on this topic can be found, e.g., in [20].

Writing the transition matrix elements in the E1 approximation (Equation 3) already suggests that some kinds of dichroism may exist, i.e., polarisation dependent absorption. In fact there are several types of X-ray dichroism such as X-ray natural linear dichroism (XNLD) [21–22] and natural circular dichroism (XNCD) [23], X-ray magnetic linear dichroism (XMLD) [24–25], X-ray magnetic circular dichroism (XMCD) [26–27], and the more exotic X-ray non-reciprocal linear dichroism [28] and magnetochiral dichroism (XMχD) [29]. In a microscopic picture, this dependence of the absorption on the polarisation of incident X-rays is caused by an anisotropy of the charge (or spin) distribution, either by bonding that yields natural dichroism or by magnetic ordering that yields magnetic dichroism. A general formulation of linear and circular dichroism was given by Carra and Altarelli [30] and an overview of the different types of dichroism can be found, e.g., in [31].

### Extended X-ray absorption fine structure (EXAFS)

In a simplistic picture, the outgoing photoelectron of the atom excited by X-rays can interfere with the backscattered waves from neighbouring atoms. That leads to the oscillatory structure in X-ray absorption spectra far above the absorption edge and was named EXAFS by Prins and Lytle [32].

Beside this microscopic picture, the EXAFS can be related, in terms of electrodynamics, to the influence of atoms close to the absorbing atom on the transition matrix elements [33]. Investigations of these influences, i.e., the scattering of the photoelectron, give the possibility to extract information about the distance and type of atoms in the vicinity of the absorbing atoms. By means of this short-range effect, EXAFS oscillations can also be obtained from non-crystalline materials in contrast to classical diffraction methods such as X-ray diffraction or electron diffraction. Regarding the case of nanoparticles, the accuracy in structural and chemical characterisation by EXAFS analysis is not lowered by line broadening, as it is in the case of diffraction methods.

The EXAFS χ(*k*) is extracted from the absorption spectrum after subtraction of a background related to the absorption of a free atom embedded in the electronic structure of the solid. Thus,

[5]
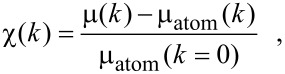


where *k* is the photoelectron wave number according to

[6]
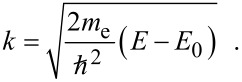


with the threshold energy *E*_0_. A theoretical framework for the EXAFS description was established in the 1970s by several groups [34–37]. Within multiple scattering theory, the EXAFS can be described as the sum of the imaginary part of contributions from different scattering paths [38–39]:

[7]
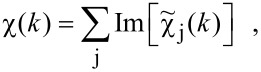


[8]
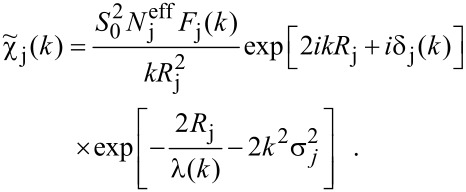


In this equation, 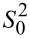 denotes an amplitude reduction factor due to many-body effects, 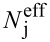 is the effective coordination number, *F*_j_(*k*) is the effective scattering amplitude, *R*_j_ is half the total of the scattering path, λ(*k*) is the mean free path length of the photoelectron wavenumber, δ_j_ is an effective total phase shift including contributions from the absorber atoms and all scattering atoms, and exp(−2*k*^2^σ_j_^2^) is the EXAFS Debye–Waller factor.

Over the last few decades, special cases of EXAFS such as its magnetic counterpart MEXAFS [40–43], as well as surface EXAFS [44–45], have also drawn much attention. In the latter case, the surface sensitivity is ensured by detecting the Auger electron emission of a particular element as a function of photon energy [36], partial FY [46–47] or total FY [48]. Also atomic EXAFS has been discussed assuming interstitial charges as scattering centres [49–51].

### EXAFS analysis

Nowadays, EXAFS analysis is usually carried out by the comparison of experimental data with calculated spectra. Its theory is implemented, e.g., in FEFF [38–39], WIEN2k [52], GNXAS [53], the Munich SPR-KKR algorithm [54] and others [55]. For a detailed analysis, quantifying lattice parameters and local chemical composition, the comparison to calculated EXAFS is indispensable. Fitting programs such as FEFFIT [56], based on the FEFF algorithm, try to find the best agreement between the calculated EXAFS and experimental data either in *k*-space or after Fourier transformation in real space. FEFFIT uses the cumulant expansion method [57–58] with the first four cumulants (Δ*R*, σ^2^, C_3_, C_4_) of the pair distribution function (PDF) of atoms around the absorber atom. To account for thermal or configurational disorder, the complex wavenumber *p* is introduced and should be used instead of *k*. The imaginary part of *p* represents losses of photoelectron coherence, which includes the mean free path and core-hole lifetime. The resulting modified EXAFS equation (Equation 8) can be written as [59]:

[9]
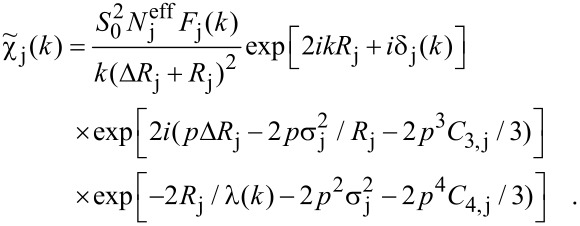


However, for each system it is essential to check whether or not it is necessary to include the fourth-order and third-order cumulants in the fitting procedure.

In contrast to the Fourier based method, a wavelet transform offers the possibility to obtain information directly about changes in the local environment of the absorbing atoms compared to a known reference sample without calculation of the EXAFS data by numerical approaches. In the following, the analysis of EXAFS on the basis of a standard Fourier transform method is described in more detail as well as wavelet transforms as a useful tool, which is rarely used for this application. Both methods first require a background subtraction from the experimental data, as already mentioned above (Equation 5). This is usually performed by using the AUTOBK algorithm [60] that approximates μ_atom_(*k*) by a spline and minimises, in a fitting procedure, the non-structural oscillations in χ(*k*). These oscillations correspond to distances in real space that are too short to be related to neighbouring atoms.

### EXAFS analysis based on Fourier transform

The discussion of the Fourier transform (FT) of experimental EXAFS data, as carried out after the pioneering work of Sayers, Lytle, and Stern in 1970 [61], is commonly employed for a structural analysis. It can be used for a quantification of the coordination number of absorbing atoms and the distance of backscattering atoms from the absorber, which is essential for any numerical EXAFS analysis on the basis of Equation 9. In addition, the elemental species of backscattering atoms have to be known for subsequent fitting of the numerical simulation to the experimental data.

**Figure 1:**
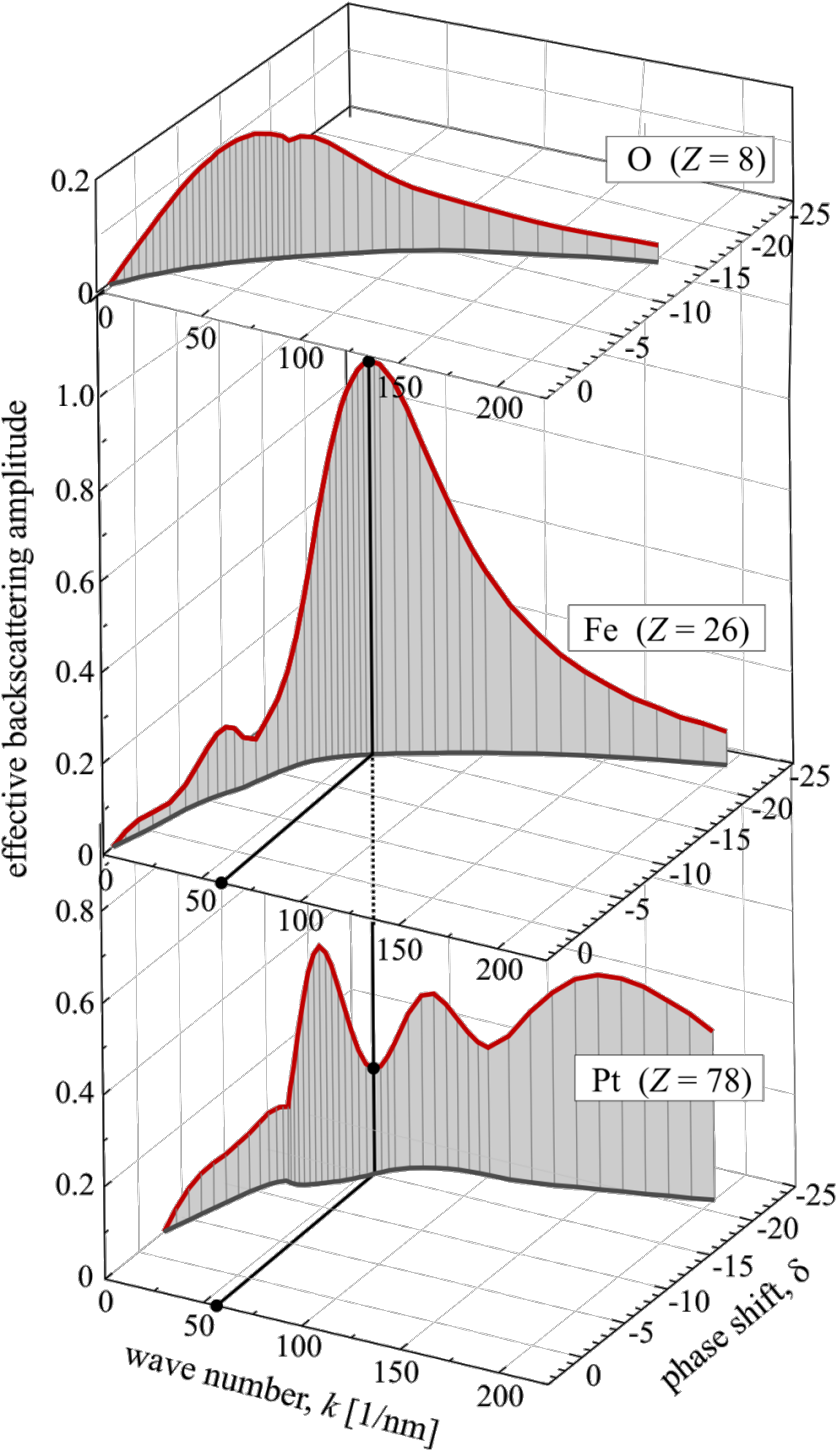
Effective backscattering amplitude of O, Fe, and Pt as a function of wave number and phase shift. At k = 60 nm^−1^ Fe has it maximum backscattering amplitude, while it exhibits a local minimum in the case of Pt due to the Ramsauer–Townsend effect.

For a proper FT of experimental data, χ(*k*) is usually weighted by *k*^n^ with n = 1, 2, 3 to compensate for the reduction of the EXAFS amplitude with increasing *k*. The value of n can give a rough estimation of the type of backscattering atom since the *k* dependence of the backscattering amplitude, and consequently, the amplitude of χ(*k*), depends on the atomic number. The lighter the element, the lower is the amplitude at high values of *k*. Examples of the *k* dependence of light and heavier atoms can be seen in Figure 1. The effective backscattering amplitude has been calculated for oxygen (*Z* = 8), Fe (*Z* = 26), and Pt (*Z* = 78); the former as one example for very light elements; Fe and Pt since experimental results on Fe*_x_*Pt_1−_*_x_* alloys will be discussed later. It can clearly be seen that the *k* dependence of the effective backscattering amplitude is unique for each element. Light elements mainly exhibit one main peak only as a function of *k*, while for heavy elements, such as Pt, the spectral shape of the backscattering amplitude may be more complicated. In the case of elements with 78 ≤ *Z* ≤ 90, a strong reduction in the backscattering amplitude over a small range connected to a more rapidly changing phase – e.g., at *k* = 60 nm^−1^ in the case of Pt –, is known in literature as the generalised Ramsauer–Townsend effect [62–63]. In a simple picture, the wavelength of the outgoing photoelectron (about 0.1 nm for *k* = 60 nm^−1^) is well-matched to the size of the scatterer. In this case, the photoelectron may tunnel through the scattering potential and the scattering cross-section vanishes leading to a dip in the backscattering amplitude at a fairly distinct wave number. By standard FT based analysis employing the FEFF code [38–39], the wave number dependent amplitude of simulated EXAFS oscillations can be fitted to experimental data by changing the local composition around the absorber atom. With this method the elemental species can be identified with a typical error of ±2 in the atomic number. Especially in chemically disordered systems of more than one element, the identification of the backscattering atoms and their distance to the absorber by this trial and error method is not efficient, and the elements have to be preselected.

**Figure 2:**
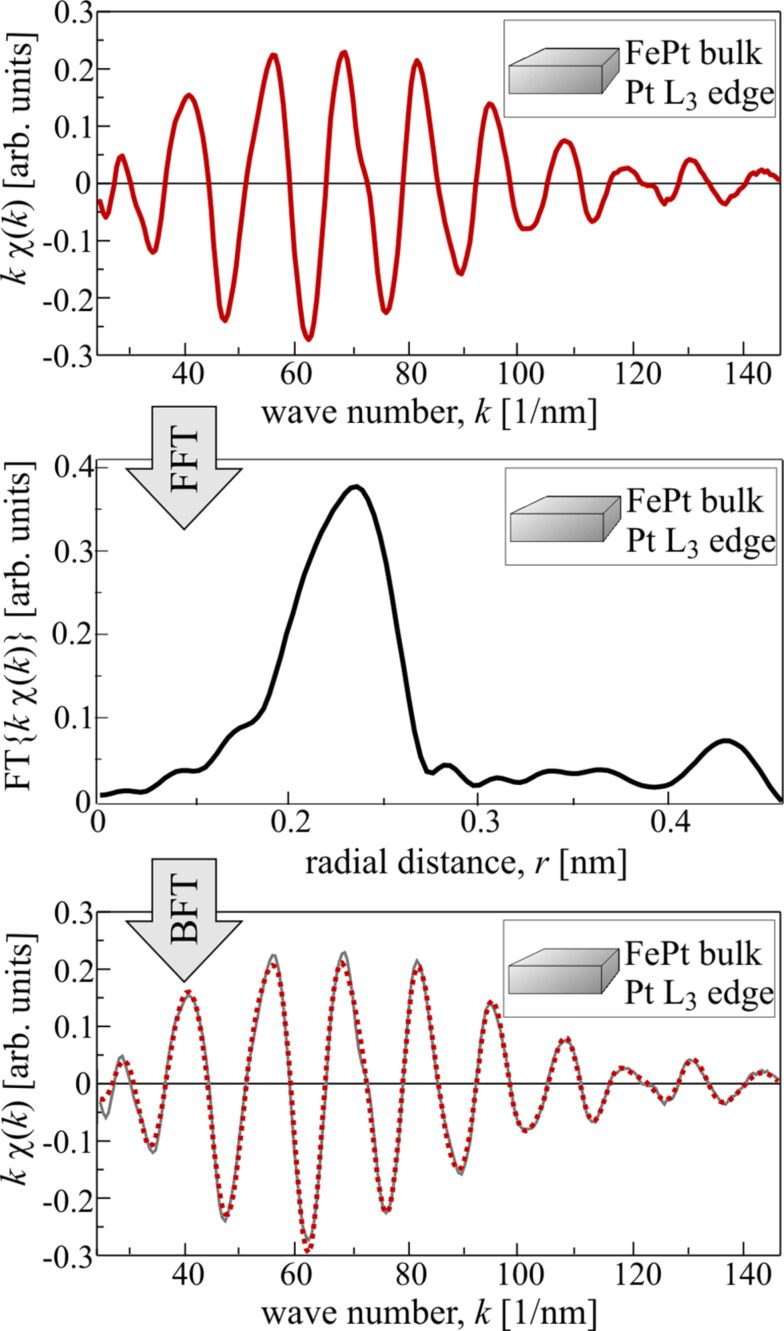
Experimental EXAFS data measured at the Pt L_3_ absorption edge of FePt bulk material at room temperature (upper panel). The forward FT (FFT) yields a pseudo-RDF (centre) that recovers (red dotted line, lower panel) the original data (grey line, lower panel) after application of a backward FT (BFT). Details on the window functions used here can be found in the text.

The real space distance of the backscattering atoms to the absorber can be estimated by an FT of the experimental χ(*k*) since it will give a pseudo-radial distribution function (RDF) of the distances of backscattering atoms. Note that this is not the geometric radial distance obtained after an FT, since the EXAFS phase shift is included in the experimental data, which yields a shift of the RDF to smaller values of the distance *r*. An example of experimental EXAFS data in *k*-space and real space is shown in Figure 2. In this case, the data have to be multiplied by a window function *W* since for low values of *k* (XANES regime) the resonant absorption dominates the scattering effects, and for high values of *k* the signal-to-noise ratio becomes too small. The data are shown only in the region of non-vanishing values of the window function. The forward FT (FFT) can be written as

[10]
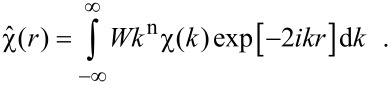


Very commonly used window functions *W* are, e.g., the Hann window (sometimes called Hanning window in the literature) and the Kaiser–Bessel window. The window should be chosen in such a way that the data interval starts and ends at a *k* value of zero-crossing of χ(*k*). The backward FT (BFT) of the pseudo-RDF should match the original data in the region where the window function leads to non-vanishing results, as shown in Figure 2 where in the lower panel the original data (grey line) are plotted together with the BFT of the FFT data (red dotted line). In this specific case a Kaiser–Bessel window has been used with a sharp truncation (d*k* = 1 nm^−1^) starting at *k* ≈ 21 nm^−1^ and ending at *k* ≈ 147 nm^−1^ for FFT, and (d*r* = 0.01 nm) starting at *r* ≈ 0.14 nm and ending at *r* ≈ 0.46 nm for BFT.

### Wavelet transforms

An obvious disadvantage of FT is that it only has a resolution in Fourier space and not in the space of original data. In EXAFS analysis, the FT magnitude of experimental data provides resolution in the radial distance of neighbouring scatterers, however, the information is lost at the wave number *k* at which the scatterer contributes. Since the position in *k*-space is related to the atomic species of the backscattering atom, important information is lost in the magnitude of the transformed signal.

It is possible to lessen this problem through the use of a short-term Fourier transform (STFT), which determines the Fourier coefficients of the original data multiplied by a window function, i.e., the *k* dependent EXAFS data are transformed in several intervals of *k*. However, this leads to a high resolution in *k* only, at the expense of good resolution in *r*, and vice versa, since cutting the signal corresponds to a convolution between the original data and the cutting window. Convolution in *k* is identical to multiplication in *r*, and since the FT of a sharp cut contains all possible values of *r*, the FT of the EXAFS data will be smeared out.

This shows that cutting the signal into several parts is the right way to obtain resolution in *k*, but the cutting has to be performed carefully so as not to lose good resolution in Fourier space. The most recent solution up to now is the wavelet transform (WT).

WTs gained much attention in the 1990s after the discovery of a family of orthogonal continuous wavelets by Daubechies [64]. Wavelets are square-integrable functions and the integral over the wavelet is zero:

[11]
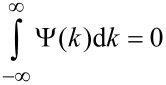


Today wavelets are widely used to extract information from audio signals and images, and for compression/decompression algorithms. However, in EXAFS analysis they are only used occasionally [65–69].

**Figure 3:**
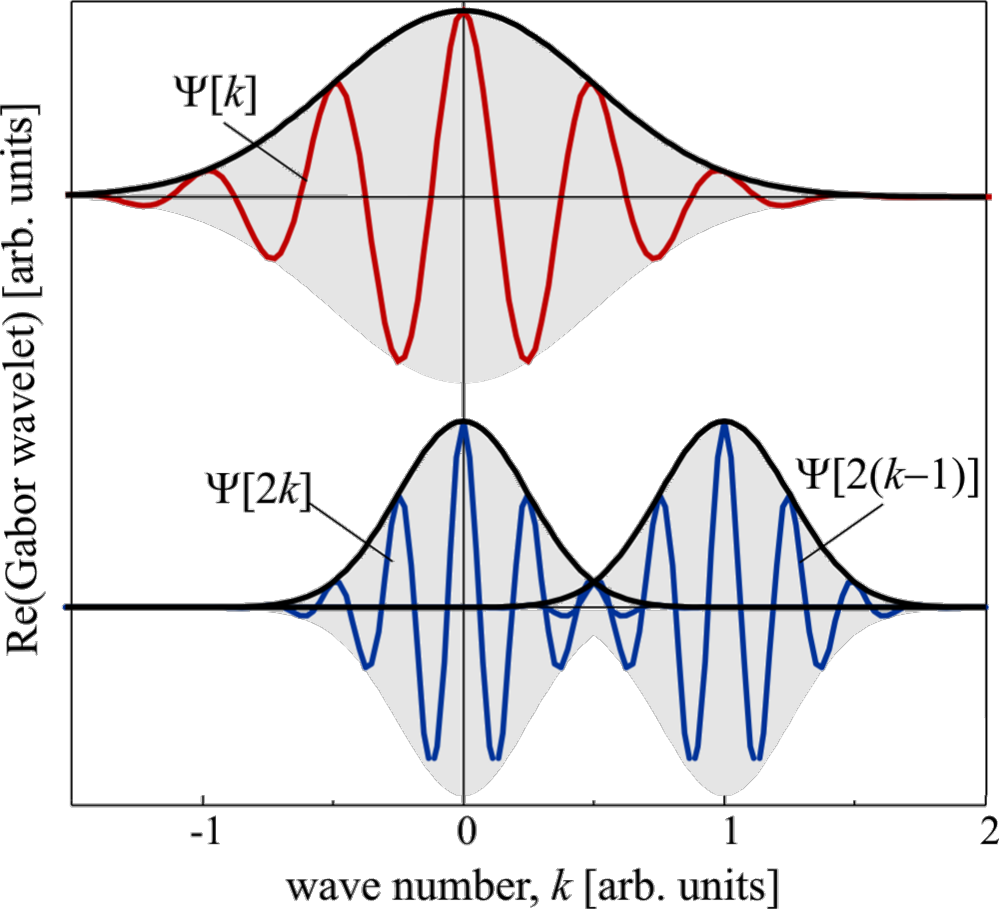
Real part of a Gabor mother wavelet (red curve) and baby wavelets generated by scaling and shifting (blue curves).

The main idea behind the wavelet transform (WT) is to replace the infinitely expanded periodic oscillations in an FT by using located wavelets as a kernel for the integral transformation: A scalable mother wavelet or analysing wavelet, Ψ(*k*), is used as a window function for the transform. The basis of the transformed signal is the so-called baby wavelets generated not only by translation but also by scaling of the mother wavelet. As an example, Figure 3 shows the real part of a Gabor wavelet that is used for EXAFS analysis, as discussed below. Starting from a mother wavelet (red curve), the first set of baby wavelets (blue curves) can easily be generated. The obtained family of wavelets can be written as

[12]
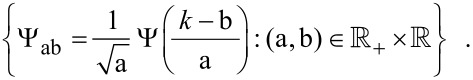


The scaling factor, a, is the most important parameter to solve the signal-cutting problem mentioned above. The wavelet as a window for the transform is shifted along the signal, and for every position the WT is calculated. This process is repeated in many cycles using scaled wavelets, i.e., wavelets with a stronger or weaker localisation in *k*, which results in a collection of WT of different parts of the original signal, with different resolutions. Merging all the information, WT yields a high resolution in both *r-* and *k*-space. Of course, similar to FT the WT is a complete transformation, i.e., the backward transformation recovers the original signal without loss of information, which is the sine qua non in data analysis. For the EXAFS analysis presented here, the Gabor wavelets have been used which have a structure similar to a typical EXAFS signal, since it consists of a slowly varying amplitude term, while the phase term is oscillating rapidly. Another similar family of wavelets that can be used for the purpose of EXAFS analysis is built from Morlet wavelets. In the work of Funke et al. [67], Morlet wavelets have been used to analyse the short-range structure of a Zn–Al layered double hydroxide. Figure 3 shows the real part of some Gabor wavelets. The WT of the EXAFS signal can be written as

[13]
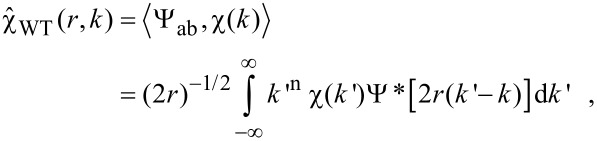


where Ψ* is the complex conjugate of the wavelet, shifting of the wavelet corresponds to b = *k* and the scaling to a = (2*r*)^−1^.

**Figure 4:**
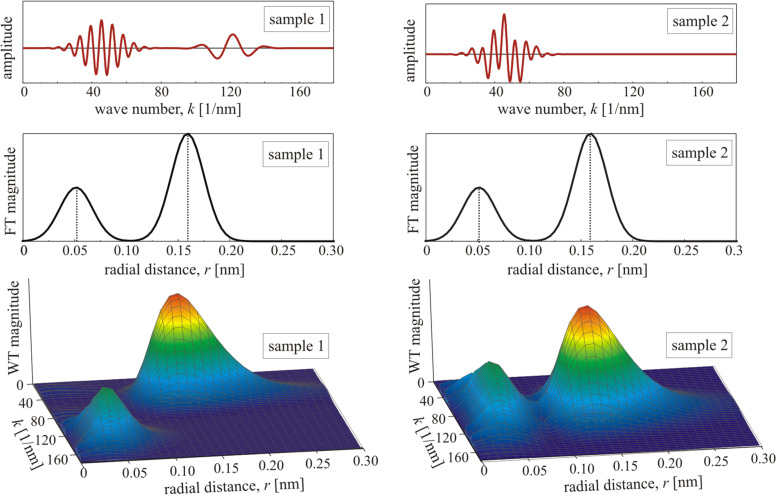
Two different sample signals (upper panel) that show the same radial distance function after FT (centre), but clearly different WT signals (lower panel).

The advantage of a WT with respect to an FT is visualised in Figure 4, where two different sample signals are plotted and analysed. The two wave packets contributing to the sample signal on the left hand side and on the right hand side of Figure 4 are the same except for their position in *k*. While in the first signal (Figure 4, left) the two packets are well-separated, they have the same position in *k* in the other signal (Figure 4, right). In the time–frequency domain it would mean that they occur at the same time for the latter case. In the reciprocal *k*-space (the real-space domain of EXAFS) it can be interpreted as a signal from two different elements with either a distinct difference in the position of maximum backscattering amplitude (first sample signal), or with the same position of maximum backscattering amplitude. Since the position of maximum backscattering amplitude is related to the atomic number of an element, as discussed before, one may state that the first sample signal describes EXAFS arising from two backscattering elements with a clear difference in their atomic number *Z*, whereas the second sample signal can be assigned to EXAFS caused by the same element (or two elements with slightly different atomic numbers).

The FT shown in Figure 4 is in both cases the same RDF, i.e., the information on the *k* position of the different wave packets is lost. In the WT the two different sample signals can still be distinguished, and both the position in *k* and the corresponding radial distance *r* can be extracted. The WT of the sample signal with well-separated wave packets shows two maxima: one located at the point (*k* ≈ 46 nm^−1^, *r* ≈ 0.16 nm) and the other at (*k* ≈ 120 nm^−1^, *r* ≈ 0.05 nm). The WT of the sample signal with coinciding wave packets also shows two maxima, but at (*k* ≈ 46 nm^−1^, *r* ≈ 0.16 nm) and (*k* ≈ 46 nm^−1^, *r* ≈ 0.05 nm).

In all cases, the values of *k* and *r* where the maxima are located are the same as those used for the generation of the sample signal. Compared to the FT of the signals, the *r* values are also the same. Since one usually does not know where the maximum in *k*-space is located for different contributions, a WT is currently the only method to receive information of the signal both in *k*-space and in real space. This is especially useful in EXAFS analysis of alloys in which different types of backscatterers are distributed on a regular lattice. A detailed discussion on the influence of rapid phase changes in EXAFS signals on the WT, and the limitations of WTs, can be found in the work of Funke et al. [67].

**Figure 5:**
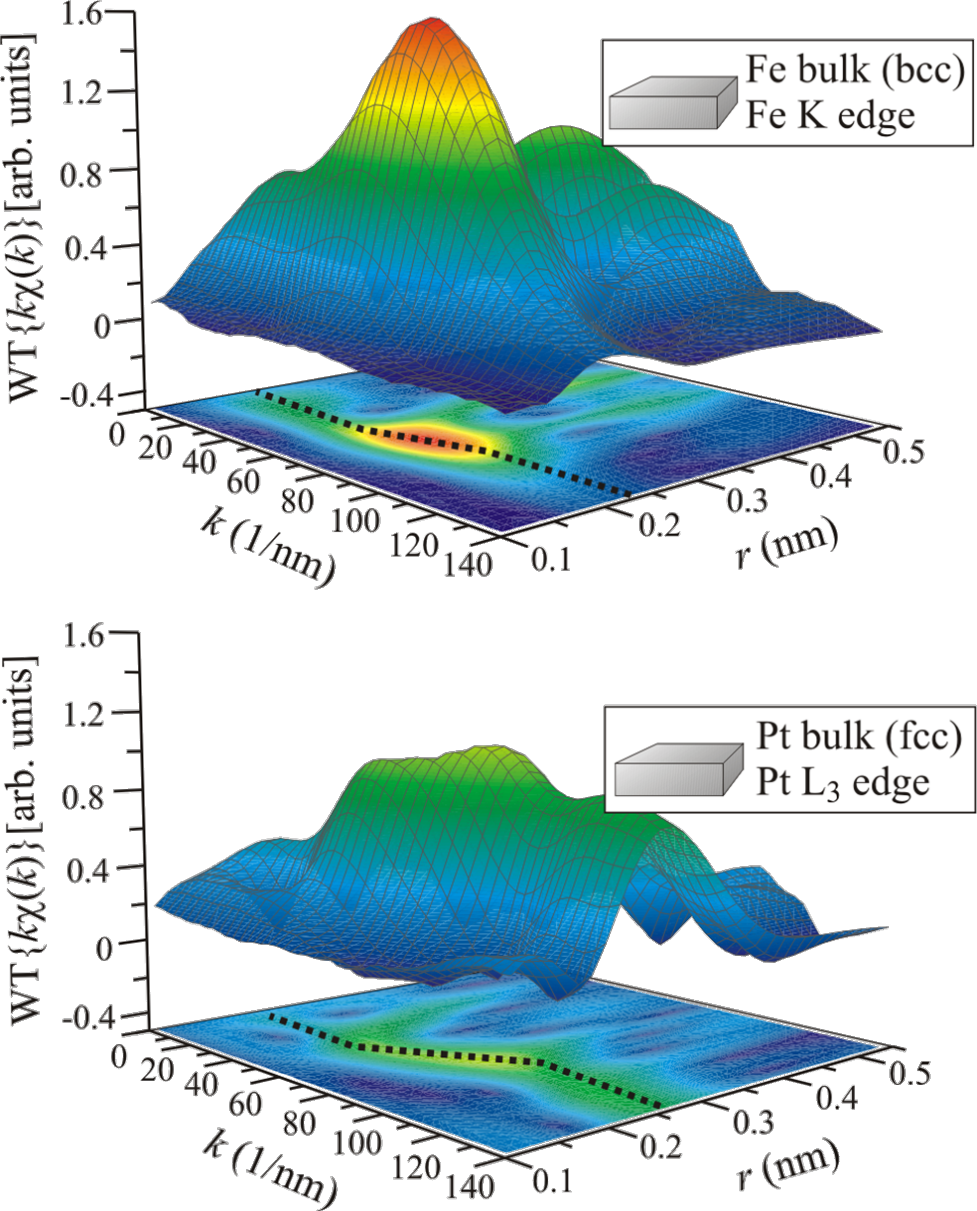
WT of room temperature EXAFS data for Fe (upper graphic) and Pt (lower graphic) reference samples measured at the Fe K and Pt L_3_ absorption edge, respectively.

The application of WT and FT to experimental data is presented in the next section through the discussion of recent results on FePt nanoparticles [68–69]. In order to facilitate the interpretation of spectral features in the EXAFS data and their WT, Figure 5 shows the WT of Fe and Pt bulk material and, for clarity, the projection of the WT on the (*k*,*r*)-plane is added. For the case of Fe, one main peak is located around *k* = 60–70 nm^−1^, as expected from the *k* dependence of the backscattering amplitude of Fe presented in Figure 1. For small *k* values the maximum is located around *r* ≈ 0.20 nm, and for large values it is located around *r* ≈ 0.23 nm. For intermediate *k* values, around the global maximum, the position in *r* of the local maxima (black dotted line in Figure 5) changes with *k* indicating a non-linear *k* dependence of the EXAFS phase shift [70]. Actually, one may consider the phase shift shown in Figure 1 for the case of Fe to change linearly for *k* < 40 nm^−1^ and *k* > 100 nm^−1^ while a distinct curvature is visible in the region in between. This is even more pronounced for the case of Pt with its rapidly changing phase in the same region of *k*. The position in *r* for the local maxima is around *r* ≈ 0.20 nm for low values of *k*, and for large values located around *r* ≈ 0.26 nm. In agreement with the *k* dependence of the backscattering amplitude shown in Figure 1, the maximum WT amplitude is reduced with respect to Fe, exhibits several peaks and has a larger magnitude at high *k* values.

### FePt nanoparticles

From the technological perspective, FePt has become one of the most interesting nanostructured materials (see, e.g., [71–74]), since its large magnetic anisotropy of 6 × 10^6^ J·m^−3^ [75–78] in the chemically ordered state with L1_0_ crystal symmetry makes it the prime candidate for new ultrahigh density storage media. The formation of the L1_0_ ordered phase is driven by volume diffusion and can be induced by post-deposition annealing of the nanoparticles or in-flight annealing of FePt nanoparticles synthesised by condensation from the gas phase before landing onto a substrate as described below.

#### Gas phase synthesis

FePt nanoparticles can be prepared by an inert gas condensation method based on a DC magnetron sputtering process from alloy targets in a continuous gas flow of helium and argon [79–80]. In general, the experimental setup for preparing nanoparticles from the gas phase is composed of three parts: A nucleation chamber, a sintering oven and a deposition chamber. After nucleation and particle growth in the nucleation chamber with liquid nitrogen cooled walls, the particles pass the sintering oven and can be in-flight annealed before deposition onto a substrate. Due to the short flight time through the sintering oven (about 1 s or less depending on the gas flow rate), annealing has to take place at very high temperatures around 1000–1300 K in order to obtain the chemically ordered L1_0_ phase in the nanoparticles. It has been shown that it is possible to prepare chemically disordered Fe*_x_*Pt_1−_*_x_* particles with diameters in the range of 3 nm < *d* < 20 nm that are single crystalline or multiple twinned with an icosahedral shape. Size and morphology can be tuned by changing the inert gas pressure and the sintering temperature [79]. It was found that the icosahedral particles are thermally stable and cannot easily be transformed into the L1_0_ phase. This indicates inadequate volume diffusion in the icosahedral particles probably due to a lack of a sufficient number of vacancies.

A method to destabilise the icosahedral shape, and to promote the formation of single-crystalline fcc FePt nanoparticles, is the introduction of oxygen during particle preparation [81]. However, the formation of the L1_0_ phase was not observed indicating that the volume diffusion is still inadequate in this method. For gas phase synthesised nanoparticles, a possible surface segregation of Pt has recently been discussed [82] as it is also known for thin FePt films [83]. In addition, an indication of a stronger lattice expansion towards the surface layer was found by analysis of transmission electron microscope (TEM) images [82]. However, since the particles were exposed to air before being transferred into the TEM, oxidation may also be responsible for the lattice expansion at the surface layers. In order to exclude the influences of oxidation and other contaminations on the investigated structure, EXAFS of in situ cleaned and oxide-free FePt nanoparticles seems to be a suitable tool to study the intrinsic structural properties of pure metallic nanoparticles.

#### Wet-chemical synthesis

A possible organometallic route to synthesise FePt nanoparticles follows the approach by S. Sun et al. [84] by the reduction of platinum diacetylacetonate, Pt(acac)_2_ and thermal decomposition of iron pentacarbonyl, Fe(CO)_5_, in hexadecane-1,2-diol at about 300 °C. The chemical reactions were initiated in the presence of the surfactants oleic acid and oleyl amine, thus providing a route to synthesise nanoparticles of a chemically disordered Fe*_x_*Pt_1−_*_x_* alloy surrounded by the surfactants. After cooling to room temperature, the particles were precipitated by adding ethanol and separated by centrifugation. After this procedure, the particles were dispersed in *n*-hexane with surfactants, precipitated out and centrifuged once again. This can be repeated several times, until a stable dispersion of nanoparticles in *n*-hexane is obtained. The nanoparticles can be brought onto a naturally oxidised Si substrate using the spin coating technique, dip coating or just by putting a small droplet of nanoparticle dispersion onto the substrate. The shell of organic ligands prevents the agglomeration of the nanoparticles and drives the formation of hexagonally self-assembled superlattices.

The quality of the hexagonal arrangement can be improved by an excess of surfactants in the dispersion. Subsequent annealing of the nanoparticles has been tried as a route to obtain nanoparticles in the L1_0_ state. Due to the thermal decomposition of the ligand shell during the annealing process and the enhanced mobility of the nanoparticles at elevated temperatures, this procedure leads to sintering of the nanoparticles especially for small diameters below 6 nm. Much effort has been taken to prevent sintering using different methods, e.g., linking of the nanoparticles to the substrate by special molecules [85–88]. Another method that has been successfully applied is the coverage of the nanoparticle monolayer, e.g., by carbon [77] or embedding in a NaCl matrix [89].

### EXAFS results and discussion

In order to gain more insight into thermally activated diffusion processes in FePt nanoparticles, it is useful to analyse the crystal structure and the homogeneity of the chemically disordered alloy. The results presented here in detail were obtained on nanoparticles prepared by the wet-chemical route described above. The magnetic properties are also compared to the properties of FePt nanoparticles synthesised by condensation from the gas phase. By EXAFS analysis it was found that there exists (i) a lattice expansion with respect to the corresponding bulk material in wet-chemically synthesised FePt nanoparticles [90] and (ii) a compositional inhomogeneity in chemically disordered nanoparticles, i.e., Fe atoms are in an Fe-rich environment and Pt atoms are in a Pt-rich environment [68–69].

**Figure 6:**
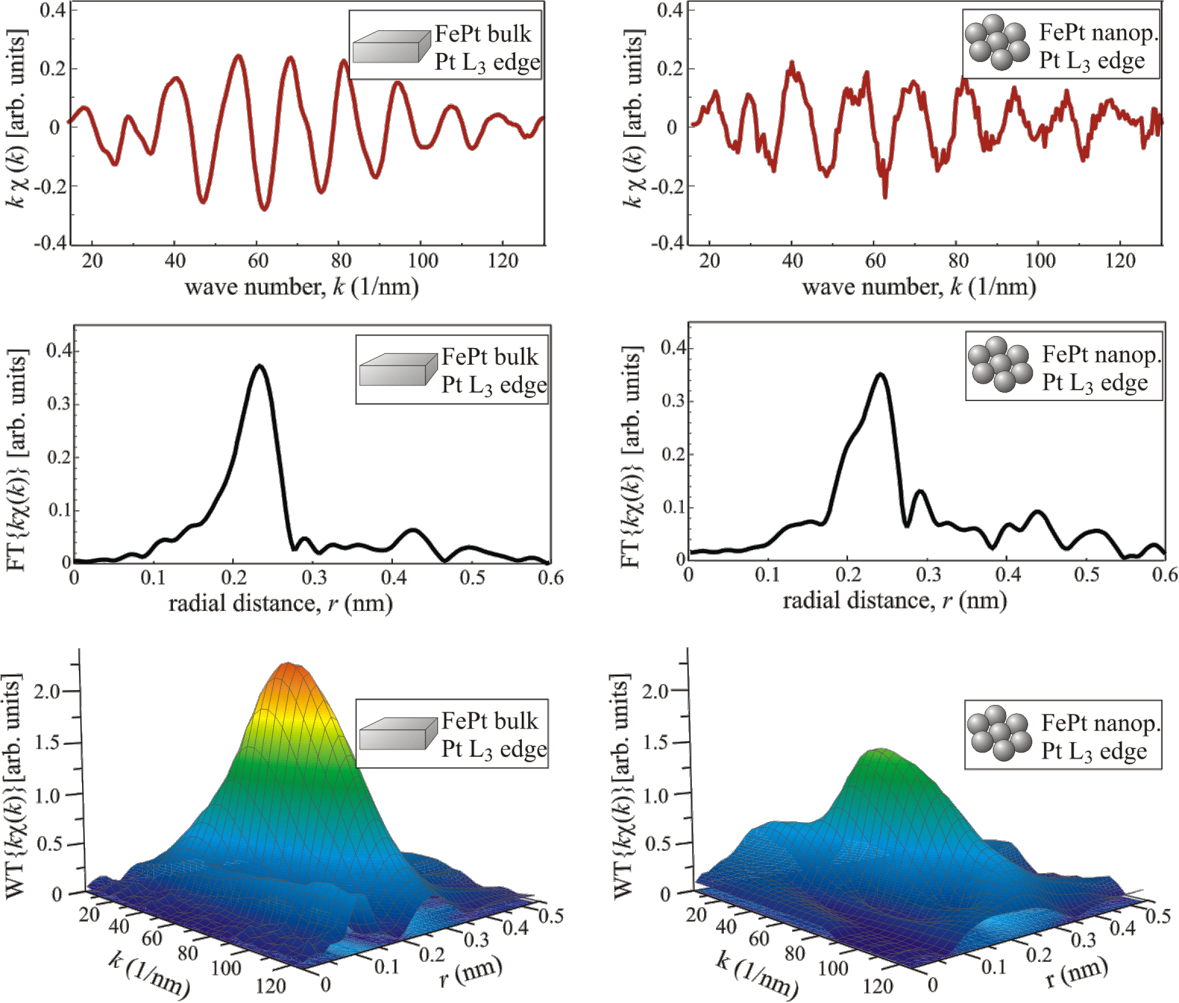
Room temperature EXAFS data of FePt bulk material (left panel) and nanoparticles (right panel) measured at the Pt L_3_ absorption edge, their Fourier transform, and wavelet transform.

Figure 6 shows the experimental EXAFS data at the Pt L_3_ absorption edge for FePt in the bulk and nanoparticulate system, respectively, and their FT and WT. While the lattice expansion can clearly be resolved [90], it is difficult to obtain small compositional changes by an FT. However, one may notice a drop of the envelope of χ(*k*) data measured at the Pt L_3_ edge of nanoparticles compared to the data obtained from bulk material and a shoulder in the FT correlated to this drop. This can be interpreted as a Pt enrichment around Pt absorbers in nanoparticles, since the backscattering amplitude of Pt exhibits a local minimum at *k* = 60 nm^−1^ reducing the amplitude of χ(k), while Fe has its maximum backscattering amplitude around this value of *k*, as can be seen in Figure 1. The differences can be seen more clearly in the WT of EXAFS data shown in the lower panel of Figure 6.

**Figure 7:**
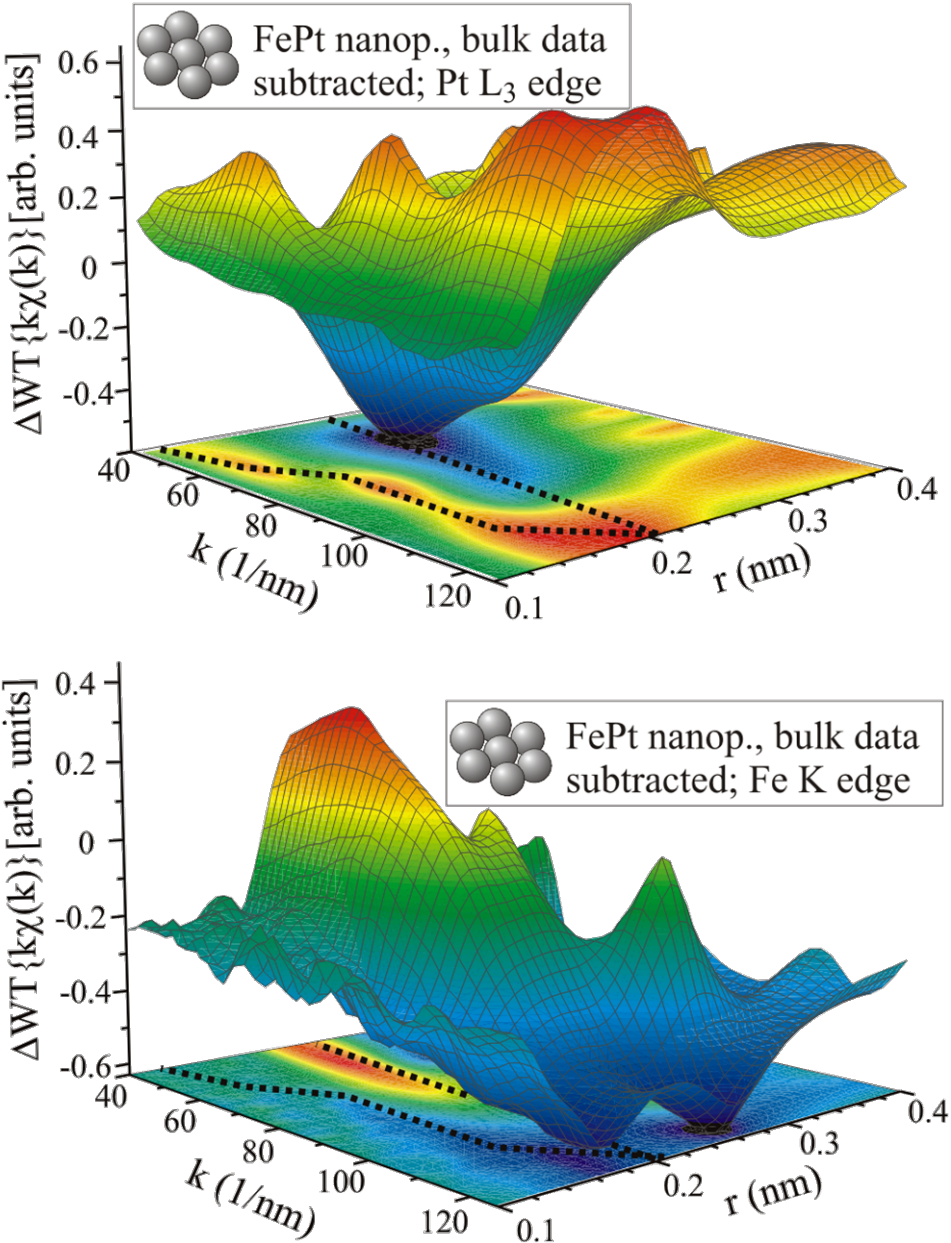
WT of room temperature EXAFS data of FePt nanoparticles measured at the Pt L_3_ (upper graphic) and Fe K absorption edge (lower graphic) after subtraction of the WT of the corresponding bulk data [68].

The WT exhibits a global maximum around *k* ≈ 60 nm^−1^ and *r* ≈ 0.22 nm for the bulk material and *k* ≈ 60 nm^−1^, *r* ≈ 0.24 nm for the nanoparticles indicating the lattice expansion in the particles. The maximum amplitude of the WT is strongly reduced for the nanoparticles and the shape differs significantly from the WT of bulk data especially in the region of low *k* values. In addition, the centroid of the plotted area is slightly shifted towards higher *k* values that may already indicate a Pt enrichment with respect to the bulk material. By comparison to the reference data shown in Figure 5, this interpretation becomes obvious: While for the FePt bulk material the WT is dominated by the sharp maximum related to Fe backscattering atoms, in the case of nanoparticles the WT is similar to the smoother WT of Pt backscattering atoms. That means that in the nanoparticles, the Pt absorber atoms are surrounded by more Pt backscattering atoms than in the bulk material, although the average composition determined by energy-dispersive X-ray spectroscopy (EDS) is the same for the two samples. This difference is not a conflict between EXAFS and EDS results, but simply reflects the fact that an averaging technique such as EDS does not allow for the detection of an inhomogeneous composition of the investigated sample, whereas the EXAFS technique does. The reason is that EXAFS stems from scattering of the photoelectron by the local surroundings of the probed atoms. Thus, it can be concluded that the Pt absorbing atoms are in a Pt-rich environment and thus, Fe atoms are in an Fe-rich environment. The latter has also been proven by similar analysis of EXAFS measured at the Fe K absorption edge as can be seen in Figure 7: After subtraction of the WT of bulk data, the difference in the number of Fe and Pt backscattering atoms becomes even more evident. (For original χ(*k*) data see [68–69].) At the Pt L_3_ edge the difference has a minimum at the position of maximum backscattering amplitude of Fe (*k* ≈ 60 nm^−1^, *r* ≈ 0.2 nm) and a maximum around the corresponding Pt position (*k* ≥ 120 nm^−1^, *r* ≈ 0.2 nm). At the Fe K edge it is the opposite way round. For clarity, in Figure 7 black dotted lines denote the position of local maxima and minima of the projected WT magnitude. Although the data clearly show the compositional inhomogeneity within the nanoparticles, a quantification of the nearest neighbour atoms of either Fe or Pt is not possible directly. However, it is useful to know for further calculations of EXAFS, e.g., by FEFF, since the structure and chemical composition have to be modelled as exactly as possible in order to get reasonable results.

The best agreement between calculated EXAFS and experimental data of nanoparticles were found assuming (40 ± 8) atom % Fe around the Pt atoms and (70 ± 12) atom % Fe around the Fe atoms and a lattice constant of (0.387 ± 0.004) nm [68–69]. For the case of the FePt bulk material, the same composition around Fe and Pt atoms within experimental errors was obtained in agreement with the averaged composition measured by EDS, (56 ± 3) atom %, and a lattice constant of (0.383 ± 0.003) nm [68–69,90].

The differences in structure and local chemical environment may also strongly affect the magnetic properties of the nanoparticles compared to the corresponding bulk material as will be discussed below.

### Discussion: Influence on magnetic properties

**Figure 8:**
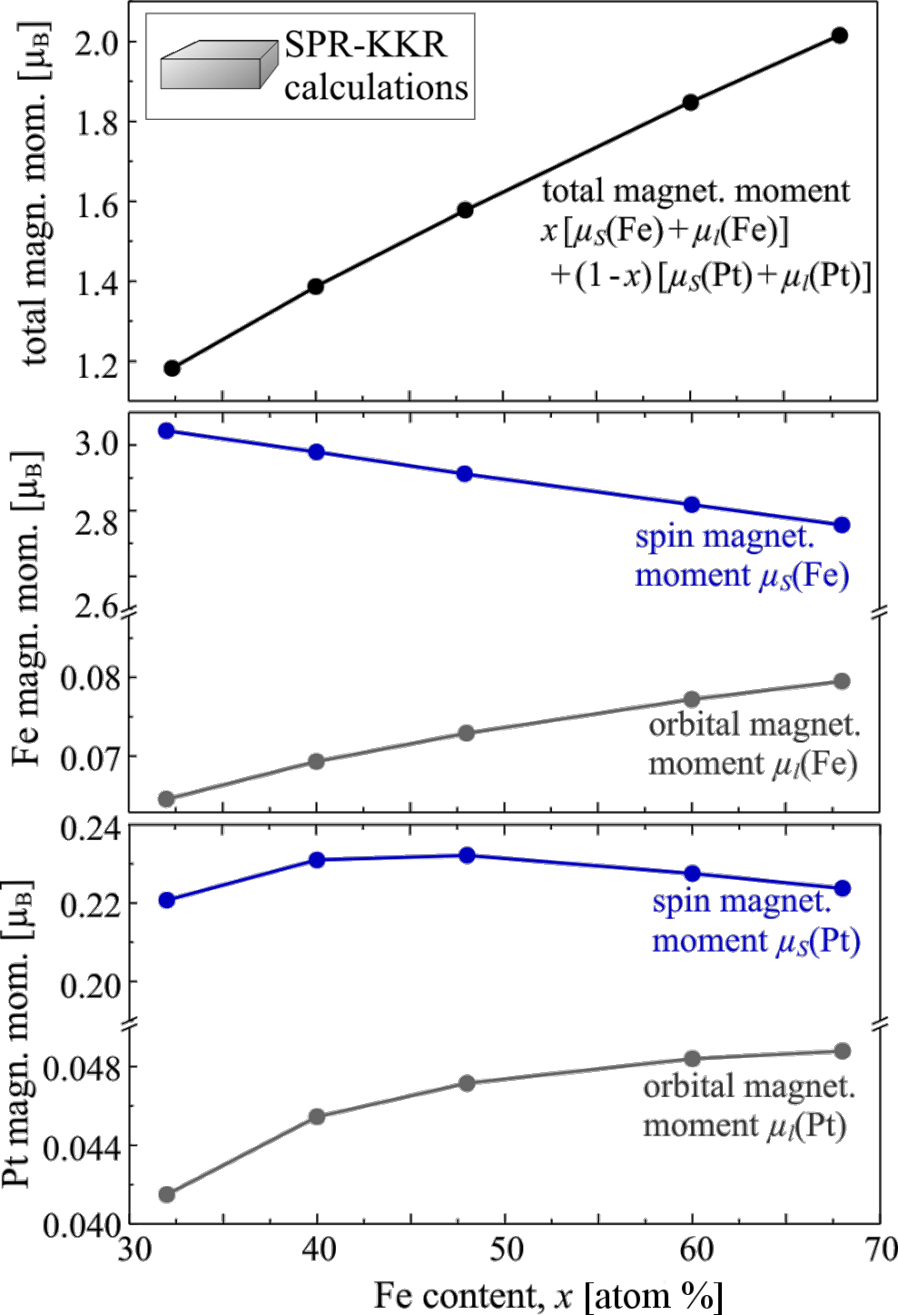
Composition dependence of spin and orbital magnetic moments at the Fe and Pt sites in chemically disordered Fe*_x_*Pt_1−_*_x_* alloys and the total magnetic moment calculated using the SPR-KKR method.

The magnetic properties strongly depend on the lattice spacing and the chemical environment around an atom. Thus, both lattice expansion and inhomogeneous composition are expected to change the magnetism of FePt nanoparticles with respect to the bulk material. The influence of the chemical environment on the magnetic moments of the Fe atoms in Fe*_x_*Pt_1−_*_x_* bulk materials have been investigated by XMCD analysis and spin polarised relativistic Korringa–Kohn–Rostoker (SPR-KKR) calculations [54,91]: The higher the Fe content in the alloy, the smaller the spin magnetic moment at the Fe sites. Changes of the orbital magnetic moment and the Pt moments are negligible with respect to the strong decrease of the Fe spin magnetic moment. The results from SPR-KKR calculations are shown in Figure 8. For the lattice constants, the experimentally determined values were used [68] as input for the calculations. It can clearly be seen that the spin magnetic moments at the Pt sites remained largely unchanged around μ_S_(Pt) ≈ 0.22 μ_B_ for different compositions between *x* = 32 atom % and *x* = 68 atom %, the orbital magnetic moment increased slightly with increasing Fe content from μ_l_(Pt) ≈ 0.042 μ_B_ to 0.048 μ_B_. The orbital magnetic moment at the Fe sites showed a similar behaviour and increased from μ_l_(Fe) ≈ 0.06 μ_B_ to 0.078 μ_B_ in the composition range investigated in this work. The Fe spin magnetic moment decreased with increasing Fe content from about 3.0 μ_B_ at *x* = 32 atom % to 2.75 μ_B_ at *x* = 68 atom %.

However, the total magnetic moment averaged over the different lattice sites increased almost linearly with increasing Fe content as is also known from experimental data using conventional magnetometry such as SQUID or VSM magnetometry [92].

Compared to experimental data on Fe*_x_*Pt_1−_*_x_* bulk-like alloys [54], it seems to be a general trend that the calculations lead to increased spin magnetic moments at the Fe sites, whereas the orbital magnetic moments are slightly underestimated. At the Pt sites the calculated values are decreased by a factor of about two, but this is in agreement with other calculated values reported in the literature [93].

The reason for this disagreement between theory and experiment is as yet unclear. However, the qualitative composition dependence is in good agreement with the experimentally obtained one.

**Figure 9:**
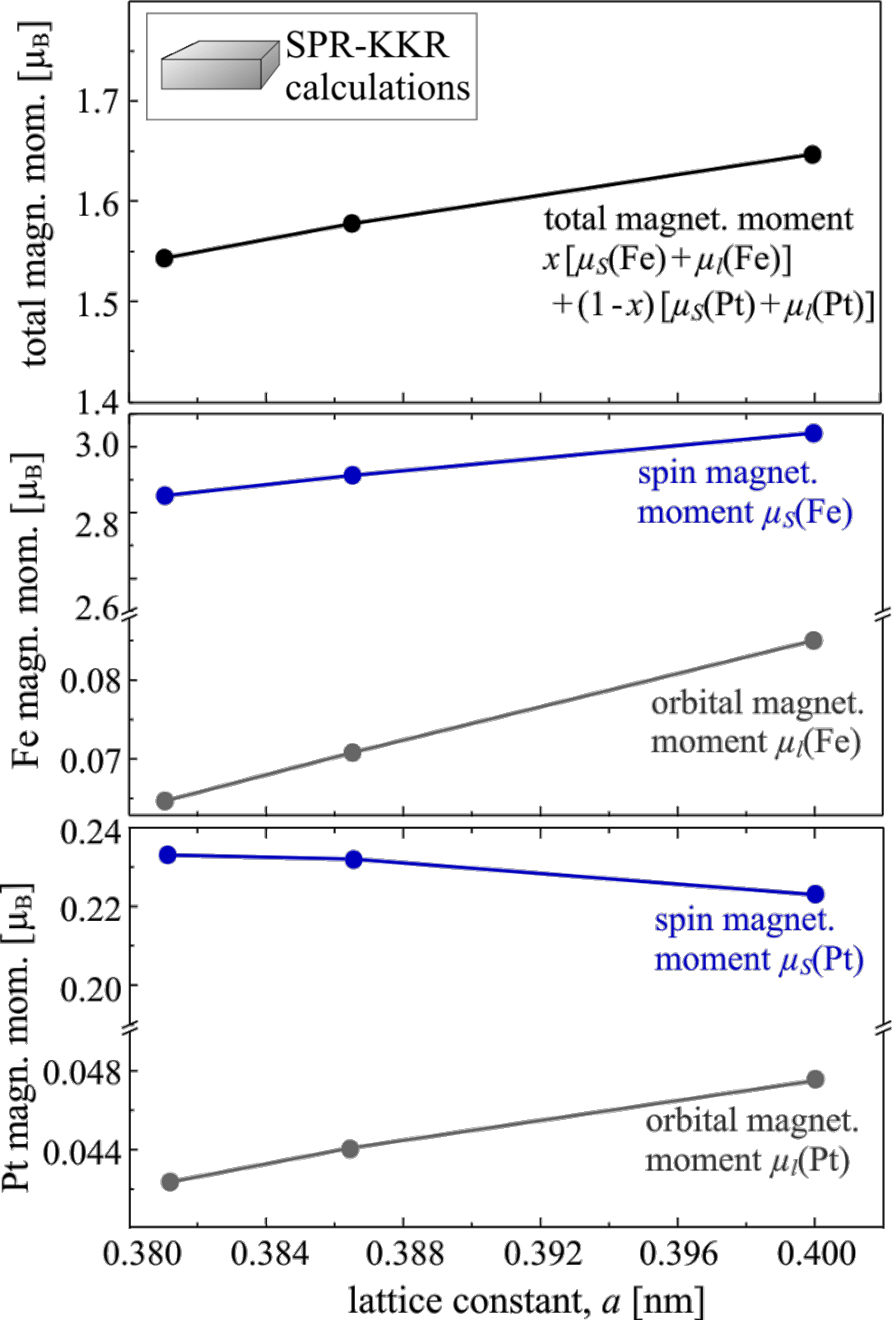
Dependence of spin and orbital magnetic moments at the Fe and Pt sites in chemically disordered FePt alloys for different lattice constants calculated using the SPR-KKR method (x = 0.5).

Regarding the effect of a lattice expansion in FePt nanoparticles, calculations offer the unique possibility to study this influence without changing any other parameter. In Figure 9, the SPR-KKR results are shown for an FePt alloy with a lattice constant of 0.381 nm as reported in the literature [94], 0.387 nm in the case of the nanoparticles, and an extremely large value of 0.4 nm corresponding to a lattice expansion of 5% with respect to the bulk value in literature. At the Fe sites, both spin and orbital magnetic moment increased with increasing lattice constant as expected in a simple picture assuming that larger lattice spacing results in a more localised electronic structure yielding more atomic-like values of magnetic moments. At the Pt sites, the orbital magnetic moment increased as well, whereas the spin magnetic moment exhibited only a slight decrease with increasing lattice constant. The total magnetic moment is larger for larger lattice parameters, but all the changes are rather small. Similar trends are reported for other ferromagnetic or antiferromagnetic materials, e.g. for bcc and fcc Fe [95]. Experimentally, the magnetic moments of FePt nanoparticles were found to be reduced with respect to the correspondent bulk material by about 20–30% [68–69].

Since the lattice expansion leads to larger magnetic moments in the order of a few percent this cannot be an appropriate explanation. However, the inhomogeneous composition can easily yield such a significant reduction of magnetic moments when the magnetic moments of nanoparticles are compared to those of bulk or bulk-like materials with the same *averaged* composition. It turns out that the magnetic moments should be assigned to the local composition around the Fe atoms since the Fe atoms, with their large magnetic moments and sensitivity to local changes, dominate the total magnetic moment.

Interestingly, reduced magnetic moments are also reported for fcc FePt nanoparticles prepared by condensation from the gas phase [96]. From this finding one may conclude that the inhomogeneity can be found in Fe*_x_*Pt_1−_*_x_* particles independent of the preparation method. The preferential formation of Fe-rich and Pt-rich regions within the nanoparticles could also influence the formation of the L1_0_ state and lower the degree of chemical order. Beside the Fe*_x_*Pt_1−_*_x_* system, a local deviation of the composition with respect to the averaged value may also occur in nanoparticles of various binary alloys.

## Conclusion

Wavelet transforms are introduced as an analysis method for EXAFS data with the potential to outperform standard Fourier based approaches especially in bimetallic alloys. The main idea behind the wavelet transform is to replace the infinitely expanded periodic oscillations in a Fourier transform by located wavelets as a kernel for the integral transformation, yielding high resolution in both real space and in *k*-space. Since the maximum backscattering amplitude exhibits different dependences on *k* for different elements, the wavelet transform visualises not only the radial distance distribution but gives also an indication of the type of backscattering atoms surrounding the absorbing atom. Thus, deviations in the local chemical environment in alloys can directly be visualised by comparison to a reference sample as it was shown for the case of FePt nanoparticles. The importance of such a detailed study of the (local) structure for data interpretation in terms of magnetic or electronic characterisation was discussed on the basis of magnetic moments of Fe*_x_*Pt_1−_*_x_* alloys measured by XMCD [68] and calculated using the SPR-KKR method. In summary, the different aspects of X-ray absorption spectroscopy, from the XANES region and its dichroism effects, to EXAFS analysis give the possibility to characterise fully nanoparticle systems regarding crystallographic and electronic structure as well as magnetic properties.
